# Examining differences in cigarette smoking prevalence among young adults across national surveillance surveys

**DOI:** 10.1371/journal.pone.0225312

**Published:** 2019-12-13

**Authors:** Peter Messeri, Jennifer Cantrell, Paul Mowery, Morgane Bennett, Elizabeth Hair, Donna Vallone

**Affiliations:** 1 Mailman School of Public Health, Columbia University, New York, NY, United States of America; 2 College of Global Public Health, New York University, New York, NY, United States of America; 3 Biostatistics, Inc., Atlanta, GA, United States of America; 4 The Schroeder Institute at Truth Initiative, Washington, DC, United States of America; 5 Department of Prevention and Community Health, Milken Institute School of Public Health, The George Washington University, Washington, DC, United States of America; 6 Department of Health, Behavior and Society, Johns Hopkins Bloomberg School of Public Health, Baltimore, MD, United States of America; National Institute of Health and Nutrition, National Institutes of Biomedical Innovation, Health and Nutrition, JAPAN

## Abstract

Accurate smoking prevalence data is critical for monitoring, surveillance, and evaluation. However, estimates of prevalence vary across surveys due to various factors. This study examines smoking prevalence estimates for 18–21 year olds across six U.S. national telephone, online and in-person surveys for the years 2013 and 2014. Estimates of ever smoking ranged from 35% to 55%. Current smoking ranged from 16% to 30%. Across the three modalities, household surveys were found to yield the highest estimates of smoking prevalence among 18 to 21 year olds while online surveys yielded the lowest estimates, and this was consistent when stratifying by gender and race/ethnicity. Assessments of the joint effect of gender, race/ethnicity, educational attainment and survey mode indicated that the relative differences in the likelihood of smoking were consistent across modes for gender and education groups. However, the relative likelihood of smoking among minority groups compared with non-Hispanic Whites varied across modes. Gender and racial/ethnic distributions for most surveys significantly differed from the U.S. Census. Over and underrepresentation of certain demographic subpopulations, variations in survey question wording, and social desirability effects may explain modality differences in smoking estimates observed in this study. Further research is needed to evaluate the effect of survey mode on variation in smoking prevalence estimates across national surveys, particularly for young adult populations.

## Introduction

Accurate smoking prevalence data is critical for monitoring, surveillance, and evaluation. However, estimates of prevalence vary across surveys due to variations in survey sampling, coverage, response rates, the effects of social desirability bias and other factors.[1] For example, online convenience samples are often recruited using non-probability based methods, leading to concerns related to coverage bias. However, more recent probability-based online samples,[2–5] lessen such concerns. Other research finds an increased risk of social desirability bias and lower reporting of sensitive behaviors for surveys involving the presence of interviewer in either in-person or telephone survey mode.[6]

Smoking prevalence estimates can also be influenced by the content and format of the assessment item. Adolescent and adult surveys often use different question wording to elicit smoking behavior, which may result in substantial differences in point estimates that are labeled *lifetime* and *current use*. Adolescent surveys typically frame *ever* or *lifetime use* as having smoked even a puff of a cigarette in one’s lifetime, while adult surveys tend to employ a more restrictive wording of the question based on having smoked 100 or more cigarettes in one’s lifetime. The *ever* or *lifetime smoking* question is then used as a filter for current use, which is framed as smoking in the past 30 days or smoking every day, some days, or not at all. The ever and past 30-day use questions are likely capturing initial smoking activity, including experimentation, initiation, and some progression, while the 100 cigarettes smoked items capture established smoking behavior.

Monitoring ever and past 30-day tobacco use among young adult populations is critical for capturing initial smoking activity among this group. Young adulthood is an important developmental period for tobacco initiation and progression to regular use. Many young people in this age range are: 1) gaining the ability to purchase tobacco legally for the first time; 2) being targeted with tobacco industry marketing[7, 8] and 3) exploring identities and seeking out new experiences, particularly related to substance use.[9] Since 2004, data finds that smoking initiation is highest among young adults age 18–21 and has not declined at the same rate as youth in recent years.[10] Recognizing this fact, states have increasingly passed Tobacco 21 laws restricting tobacco sales to those aged 21 and over[11] and recent initiatives have focused on similar legislation at the federal level.[12] Given these trends, it is especially important to understand and monitor this 18–21 year old age group separate from other age groups.

The current study adds to the literature on whether and to what extent survey modality influences estimates of smoking prevalence by addressing the following questions:

1. What are the point estimates of ever and current (past 30-day) smoking for 18–21 year-olds in the U.S. from six national surveys administered during the same time period?
2. How do point estimates vary by survey mode overall and among subpopulations?

We report on the variation in sample estimates of ever and past 30-day smoking among respondents aged 18–21 across six national surveys administered between 2013 and 2014. We examine the role of sample representativeness and assess absolute and relative differences in prevalence estimates overall and among demographic subgroups by modality. As part of this analysis, we also test a hypothesis regarding modality. To the extent that the online survey is perceived as a more anonymous modality than either telephone or in-person surveys and thus may improve reporting of sensitive behaviors [1, 13, 14], we hypothesize that the online modality will reduce underreporting of smoking compared to other modalities and therefore result in higher estimates of smoking than either telephone or in-person interviewing.

## Methods

### Data sources

Data consisted of six national probability-based U.S. surveys conducted between 2013 and 2014 (see Table 1 for details). The online surveys were Truth Initiative’s 2014 Truth Longitudinal Cohort (TLC) [2] and the 2013 Young Adult Cohort (YAC) [3, 15]; the two in-person computer assisted interviewing (CAI) surveys were the Substance Abuse and Mental Health Administration’s (SAMHSA) 2013 and 2014 National Surveys of Drug Use and Health (NSDUH) (available at https://datafiles.samhsa.gov/study-series/national-survey-drug-use-and-health-nsduh-nid13517); the two telephone surveys were from the Centers for Disease Control and Prevention (CDC) National Adult Tobacco Survey (NATS) from 2012–2013 and 2013–2014 (available at https://www.cdc.gov/tobacco/data_statistics/surveys/nats/index.htm). These six surveys were selected because they measured ever smoking in the same way with the question of whether one had ever smoked even one or part of a cigarette (see Table 2 for question wording). The telephone and in-person CAI survey data were available publicly online and we obtained question wording and the final response rate from online technical documentation. Online survey data, question wording, and response rates were provided by the Truth Initiative co-authors. All data included probability sampling weights. Data for the NSDUH and NATS surveys were publicly available and were exempt from Institutional Review Board (IRB) review. Chesapeake IRB approved data for the TLC (#20036–017) and the YAC (#20036–020).

**Table 1:** Sample characteristics of six national probability surveys of smoking conducted between 2013 and 2014.

	Modality	Sample Size AGES 18 to 21	Response Rate^1/^
Young Adult Cohort (YAC) Wave 6 2013	Online	800	6.3%^2/^
Truth Longitudinal Cohort (TLC) 2014	Online	7,585	52.5%
National Adult Tobacco Survey (NATS) 2012–2013	Telephone	1,894	45%
National Adult Tobacco Survey (NATS) 2013–2014	Telephone	2,561	36%
National Survey of Drug Use and Health (NSDUH) 2013	In-person CAI	8,929	73%
National Survey of Drug Use and Health (NSDUH) 2014	In-person CAI	6,457	71%

^1/^Response rates are for the entire sample frame of the surveys. Response rates specific to 18 to 21 age group were not ascertained.

^2/^This sample was recruited from the Knowledge Networks Panel, thus the response rate reflects the cumulative response rate (CUMRR1). The panel recruitment rate (RECR) for this sample was 14.7%, the profile rate (PROR) was 65.5% and the completion rate was 65.7%. The CUMRR1 is the product of these three rates.

**Table 2:** Estimates of ever and past 30-day smoking for 18 to 21 year old respondents in six U.S. national surveys conducted between 2013 and 2014.

SURVEY (MODALITY)	EVER USE QUESTION WORDING^a^	% EVER SMOKING	PAST 30-DAY USE QUESTION WORDING	% PAST 30-DAY SMOKING
Young Adult Cohort Study 2013 (Online)	Which of the following have you ever used or tried (even 1 puff)^b^ …?	35% (32%,38%)	Which of the following products have you used in past 30 days^b^….	17% (15%, 20%)
Truth Longitudinal Cohort 2014 (Online)	Have you ever tried smoking (even 1 or 2 times)?	39% (38%, 41%)	During the past 30 days…on how many days did you use cigarettes (even 1 or 2 puffs)?^c^	16% (15%, 17%)
NSDUH 2013 (In-person CAI)	Have you ever smoked part or all of a cigarette?	55% (53%,56%)	During the past 30 days, that is since____ on how many days did you smoke part or all of a cigarette? What is your best estimate of the number of days?^c^	28% (27%, 29%)
NSDUH 2014- (In-person CAI)	Have you ever smoked part or all of a cigarette?	49% (47%,51%)	During the past 30 days, that is since_____ on how many days did you smoke part or all of a cigarette? What is your best estimate of the number days?^c^	26% (24%,.27%)
National Adult Tobacco Survey 2012–2013 (Telephone)	Have you ever tried cigarette smoking, even one or two puffs	46% (43%,48%)	Do you now smoke cigarettes every day, some days not at all?^d^	20% (18%,22%)
National Adult Tobacco Survey 2013–2014 (Telephone)	Have you ever tried cigarette smoking, even one or two puffs	49% (47%,51%)	Do you now smoke cigarettes every day, some days not at all?^d^	19% (18%, 21%))
Test of inter-survey differences in sample estimates		F(5, 28608) = 24 p < .001	Test of inter-survey differences in sample estimates	F(5, 28185) = 45.8 p < .001

Sampling weights applied to all survey estimates and complex sampling design variables for the NSDUH surveys.

^a^ For all surveys except the YAC 2013, question responses include *yes* and *no*. Respondents who reported *yes* were coded as ever users.

^b^ Ever and past 30-day smokers defined as those who checked “yes” for cigarettes. Cigarette use was asked in the context of multiple tobacco products and product response options were randomized so as to eliminate order effects.

^c^ Past 30-day smokers defined as those who reported smoking at least 1 day in the past 30 days.

^d^ Past 30-day smokers defined as those who reported using cigarettes *some days* or *every day*.

The two online surveys used address-based sampling (ABS) based on the USPS Delivery Sequence File of all households in the U.S. Respondents were recruited primarily via mailings and data collection occurred online. The YAC cohort was based on the Knowledge Networks panel, which is primarily an ABS, probability-based random sample panel that provides statistically valid representation of the U.S. population, including cell-phone only households, African Americans, Latinos and young adults. Knowledge Networks provides households without internet access with a free netbook computer and internet service. For this particular cohort, a representative sample of young adults age 18–34 were drawn from the panel, and only one panel member per household was selected at random to be part of the study sample and no members outside the panel were recruited. The TLC cohort was a custom ABS sample of youth and young adults ages 15–21 years recruited through ABS sampling supplemented with auxiliary data to enhance recruitment success. Individuals without home access to the Internet were incentivized at higher levels than those with home access. Additional information regarding these cohorts is available elsewhere.[2, 3, 15]

The other surveys used geographic cluster sampling (for NSDUH) or random digital dial (RDD) based on combined landline and cell phone frames (for NATS). We included repeated surveys for NSDUH and NATS to increase the number of surveys available for analysis and to assess variability of estimates within the same series of surveys.

### Study measures

The main outcome measures for this study included ever smoking and current smoking among 18–21 year olds. Table 2 specifies coding for these two items for each of the six surveys. We also included demographic variables for gender (male/female), race/ethnicity (non-Hispanic White/non-Hispanic Black/Hispanic/non-Hispanic Other), and educational attainment (no high school/still in high school/high school only/some college or technical training/college degree or post-college). Surveys were classified as either online, telephone, or in-person CAI administration. The NSDUH used a combination of in-person CAI methods involving the collection of demographic information through computer assisted personal interviewing (CAPI) and tobacco use information through audio computer-assisted self-interviewing (ACASI).

### Analysis plan

For each survey, we estimated weighted means for ever and past 30-day smoking rates for 18–21 year-old respondents, as well as 95% confidence intervals for the means based on their standard errors. We conducted an F-test to assess inter-survey differences in smoking rates across samples. To assess sample representativeness and possible coverage bias, we compared unweighted demographic estimates for gender and race/ethnicity for each survey with those from the U.S. Census using a Wald chi-square test for goodness of fit to a known distribution.

Weighted least squares regression analysis was used to estimate modality-specific smoking rates and test for statistically significant modality effects across all surveys and within gender and racial/ethnic subpopulations. Because modality effects are properties of surveys rather than respondents, we treated the survey as the unit of analysis (n = 6) in these analyses. We estimated regressions in which estimates of ever and past 30-day smoking prevalences for each survey were the outcomes. The modality of the survey was the only independent variable. We suppressed the constant term in these regressions, and specified three indicator variables—one for each survey model. Thus the three coefficients for the regression model were direct estimates of the weighted ever and past 30-day smoking rates for each modality with their associated confidence interval. F statistics were obtained from a weighted regression analysis that included a constant term (online surveys were the excluded category for obtaining the F tests). The weights were the inverse of the variance for the sample estimates of the smoking rates. These analyses were conducted for the complete samples and for subgroups stratified by race/ethnicity and gender.

Next, we pooled micro-level data for surveys with the same modality and examined differences in ever and past 30-day smoking rates by gender, race/ethnicity, and education across survey modalities. We used the final population sample weights for each survey to weight the pooled sample. Sample design variables were also applied to the NSDUH surveys. There was very little missing data on any variable (<1%), thus we excluded observations with missing data for each analysis as appropriate.

Analysis of the NSDUH and NATS surveys were exempt from human subjects review as data was obtained from public archival datasets in which personal identifying information was removed. Analyses for the TLC and YAC surveys were approved by Chesapeake Institutional Review Board. We used Stata 13.0 for all analyses.

## Results

Table 1 presents modality of survey administration, sample sizes, and response rates for the six surveys. Table 2 presents question wording, weighted point estimates, and 95% confidence intervals for ever and past 30-day rates of cigarette smoking.

The range in weighted point estimates for ever smoking is approximately 20 percentage points, from a low of 35% to a high of 55% (Table 2). This range exceeds variation that would be expected by sample variation alone. Ever use elicited from the two online surveys are at the lower end of sample estimates, at 39% for the TLC and 35% for the YAC. At the other extreme, the 2013 NSDUH in-person CAI survey has the highest weighted estimate of ever use at 55%. The confidence intervals for the highest and lowest estimates do not overlap with each other or with the mid-range estimates. The estimates for the 2012–2013 and 2013–2014 NATS telephone surveys and the 2014 NSDUH in-person CAI survey occupy a middle ground. The weighted sample estimates for ever use for these three surveys are 46%, 49%, and 49%, respectively, and have overlapping confidence intervals. The narrow range of sample values for these three surveys suggests they estimate similar population values for ever use, but they differ from the lower estimates for the two online surveys and the higher estimate for the 2013 NSDUH in-person CAI survey.

Rates of past 30-day smoking display a modality effect that is similar to that for ever smoking. The two online surveys are associated with the lowest weighted estimates of past 30-day use at 16% (for the TLC) and 17% (for the YAC), the two NSDUH in-person CAI surveys are the highest estimates (26% and 30%), and the estimates for the two NATS telephone surveys fall in the middle (19% and 20%). Within each series the more recent survey estimated lower current smoking for the more recent of the paired survey. This is consistent with the general decline in current smoking rates over time found in other national surveys.

Table 3 presents unweighted demographics for gender and race/ethnicity for each survey compared with 2014 U.S. Census statistics to assess coverage and representativeness of the sampling frames for each survey. Gender distributions for each survey are significantly different from the Census with the exception of NSDUH 2014. The online samples overrepresent females and the telephone surveys overrepresent males. However, all estimates are within six percentage points of census estimates. The unweighted race/ethnic sample distributions show somewhat greater deviation from Census statistics. The online surveys overrepresent non-Hispanic Whites and underrepresent non-Hispanic Blacks, while the phone surveys underrepresent Blacks but overrepresent those reporting other race/ethnicity. In contrast, both NSDUH in-person surveys most closely approximate the Census data.

**Table 3:** Coverage estimates of U.S. gender and race distribution for respondents 18–21 (unweighted).

	% Male	% Female	M/F sample estimate is significantly different from census	% Non-Hispanic White	% Non-Hispanic Black	% Hispanic	% Other	Race/ethnicity estimate is significantly different from census
YAC 2013	45	55	*	64	8	19	9	**
TLC 2014	45	55	*	68	10	13	10	**
NSDUH 2013	49	51	*	56	14	20	10	**
NSDUH 2014	50	50		53	13	22	12	**
NATS 2012–2013	55	45	*	55	8	20	17	**
NATS 2013–2014	56	44	*	58	12	20	10	**
**U.S. Census 2014^a^**	**51**	**49**		**55**	**16**	**21**	**8**	

^a^Source: Annual Estimates of the Resident Population by Sex, Single Year of Age, Race Alone or in Combination, and Hispanic Origin for the United States: April 1, 2010 to July 1, 2016. U.S. Census Bureau, Population Division. https://factfinder.census.gov/faces/nav/jsf/pages/index.xhtml Release Date: June 2017.

*p<0.05

** p<0.001

Table 4 presents analyses examining modality effects of ever and past 30-day smoking stratified by gender and race/ethnicity. Among all race/ethnic and gender groups combined, the pooled in-person CAI surveys yield the highest weighted estimate of ever smoking at 52%, followed closely by the telephone surveys at 48% and the considerably lower online survey estimate at 39% (F(2,3) = 22.70 p<0.05). This ordering of modalities is present for gender-specific estimates when race/ethnic groups are combined. A similar pattern of modality differences is evident among non-Hispanic Whites of both genders, but is greatly diminished for racial/ethnic minority groups. Specifically, modality has no significant effect on estimated rates of ever smoking for non-Hispanic Blacks, Hispanics, and non-Hispanic Others (F-tests nonsignificant p>0.05), with the exception of significant differences across modalities for non-Hispanic Other males.

**Table 4:** Weighted least squares sample estimates and 95% confidence intervals of ever and past 30-day smoking for 18 to 21 year old respondents by survey modality, gender and race/ethnicity for six U.S. national surveys.

	GENDER COMBINED		MALE		FEMALE	
RACE/ETHNICITY	*Ever Smoking*	*Past 30-day Smoking*	*Ever Smoking*	*Past 30-day Smoking*	*Ever Smoking*	*Past 30-day Smoking*
**All Groups**						
In-person CAI Surveys	0.50 (0.47, 0.54)	0.27 (0.25, 0.29)	0.54 (0.50, 0.59)	0.32 (0.30, 0.35)	0.46 (0.43, 0.48)	0.22 (0.19, 0.25)
Telephone Surveys	0.48 (0.42, 0.54)	0.19 (0.16, 0.23)	0.52 (0.44, 0.61)	0.23 (0.19, 0.26)	0.42 (0.30, 0.55)	0.16 (0.10, 0.21)
Online Surveys	0.39 (0.34, 0.43)	0.16 (0.14, 0.29)	0.43 (0.36, 0.50)	0.19 (0.17, 0.22)	0.34 (0.31, 0.37)	0.13 (0.10, 0.16)
F(2,3)	22.70*	59.68**	10.58*	81.29**	42.47**	22.02*
**Non-Hispanic White**						
In-person CAI Surveys	0.56 (0.54, 0.59)	0.32 (0.31, 0.34)	0.60 (0.56, 0.64)	0.37 (0.35, 0.38)	0.53 (0.50, 0.56)	0.27 (0.24, 0.31)
Telephone Surveys	0.49 (0.44, 0.54)	0.21 (0.19, 0.24)	0.52 (0.45, 0.60)	0.23 (0.21, 0.26)	0.45 (0.39, 0.50)	0.19 (0.13, 0.25)
Online Surveys	0.37 (0.34, 0.41)	0.17 (0.15, 0.18)	0.42 (0.37, 0.47)	0.19 (0.17, 0.20)	0.33 (0.30, 0.36)	0.15 (0.11, 0.18)
F(2,3)	103.30**	215.50**	38.64**	413.73**	111.35**	39.35**
**Non-Hispanic Black**						
In-person CAI Surveys	0.39 (0.29, 0.48)	0.19 (0.16, 0.22)	0.41 (0.32, 0.49)	0.23 (0.20, 0.27)	0.31 (0.27, 0.35)	0.14 (0.08, 0.20)
Telephone Surveys	0.39 (0.25, 0.53)	0.13 (0.07, 0.18)	0.45 (0.27, 0.64)	0.14 (0.07, 0.21)	0.33 (0.24, 0.42)	0.11 (-0.01, 0.23)
Online Surveys	0.38 (0.28, 0.48)	0.16 (0.11, 0.20)	0.41 (0.32, 0.49)	0.19 (0.13, 0.25)	0.34 (0.28, 0.41)	0.12 (0.03, 0.21)
F(2,3)	0.33	6.17	0.24	7.25**	1.22	0.29
**Hispanic**						
In-person CAI Surveys	0.49 (0.45, 0.53)	0.23 (0.20, 0.26)	0.55 (0.47, 0.62)	0.29 (0.21, 0.37)	0.43 (0.35, 0.50)	0.16 (0.13, 0.20)
Telephone Surveys	0.51 (0.43, 0.60)	0.19 (0.14, 0.25)	0.55 (0.42, 0.68)	0.25 (0.11, 0.38)	0.47 (0.30, 0.63)	0.12 (0.05,0.18)
Online Surveys	0.45 (0.45, 0.53)	0.17 (0.12, 0.21)	0.50 (0.36, 0.64)	0.22 (0.09, 0.36)	0.41 (0.29, 0.53)	0.12 (0.08,0.16)
F(2,3)	1.72	6.70	0.53	0.92	0.44	4.47*
**Other**						
In-person CAI Surveys	0.40 (0.36, 0.44)	0.18 (0.14, 0.22)	0.43 (0.40, 0.45)	0.20 (0.17, 0.22)	0.37 (0.29, 0.44)	0.16 (0.07, 0.25)
Telephone Surveys	0.43 (0.36, 0.50)	0.16 (0.10, 0.23)	0.52 (0.48, 0.56)	0.21 (0.17, 0.25)	0.33 (0.20, 0.47)	0.10 (-0.03, 0.24)
Online Surveys	0.31 (0.26, 0.37)	0.12 (0.08, 0.17)	0.39 (0.35, 0.43)	0.14 (0.11, 0.18)	0.25 (0.16, 0.33)	0.09 (-0.00, 0.19)
F(2,3)	11.07*	4.06	39.00*	11.17*	5.82	1.45

*P < .05

**P < .01 Unit of analysis is the survey.

F-statistic tests for modality difference in sample estimates of ever and past 30-day smoking rates.

For past 30-day smoking, the qualitative pattern of modality effects is generally similar to that for ever smoking. When data are pooled across race/ethnicity and gender, the pooled sample estimate of past 30-day smoking among respondents for in-person CAI surveys is 28% followed by 19% for telephone surveys and 16% for online surveys, and these differences are statistically significant (p<0.05). This same pattern is seen among all males and females and among non-Hispanic Whites overall. Among Whites, in-person CAI survey estimates for past 30-day smoking are approximately double those obtained from online surveys. In contrast, modality effects for past 30-day smoking are greatly diminished among racial/ethnic minorities. Two exceptions to the trend of small modality effects for most racial/ethnic minorities is a significantly higher past 30-day smoking rate for in-person CAI surveys compared with online and telephone surveys among non-Hispanic Black males (p<0.01) and Hispanic females (p<0.05).

Table 5 presents results of modality-specific logistic regressions that estimate the joint effects of gender, race, and past or current educational attainment on ever and past 30-day smoking. This table presents the regression coefficients (adjusted logged odds ratios) of ever smoking estimated separately for online, telephone and in-person CAI surveys. All three modalities consistently estimate negative gender coefficients that are statistically significant (p<0.01). Furthermore, the point estimates of the gender coefficients are similar and fall within the 95% confidence intervals computed for each modality-specific regression equation. However, modality effects are evident for the race/ethnicity estimates. The ever smoking regression estimates for non-Hispanic Blacks are significantly lower than non-Hispanic Whites for the telephone and in-person CAI modes, indicating lower ever smoking among non-Hispanic Blacks compared with Whites. There is no difference between non-Hispanic Black and non-Hispanic White estimates for the online mode. Each modality yields different results when comparing non-Hispanic White and Hispanic respondents. Compared to non-Hispanic Whites, Hispanics report significantly higher ever smoking on the online surveys (p<0.01), no difference on the telephone surveys and a significantly lower rate on the in-person CAI surveys (p<0.01). Across all three modalities, sample estimates of ever smoking for the “Other” ethnic category are consistently lower than Whites at the p<0.05 level for online and in-person CAI surveys and at the p<0.10 for telephone surveys. Moreover the point estimates for the “Other” online (-0.25) and telephone (-0.21) coefficients as well as their confidence intervals are similar and substantially different from the in-person CAI estimate (-0.68). Estimates of ever smoking are lower in all three survey modalities among individuals with higher educational attainment compared with respondents not completing high school/still in high school. However, these educational differences are not statistically significant for online surveys when comparing those not completing H.S. with H.S. graduates and college graduates.

**Table 5:** Weighted logistic regression coefficients for U.S. national ever and past 30-day smoking for 18–21 respondents, surveys pooled by modality.

	Online Surveys^a^ (N = 2)		Telephone Surveys^a^ (N = 2)		In-person CAI Surveys^b^ (N = 2)	
	*Ever Smoking*	*Past 30-day Smoking*	*Ever Smoking*	*Past 30-day Smoking*	*Ever Smoking*	*Past 30-day Smoking*
Gender-Female	-0.37** (-0.50,-0.25)	-0.41** (-0.58,-0.23)	-0.35** (-0.51,-0.20)	-0.43** (-0.63,-0.23)	-0.34** (-0.44,-0.24)	-0.47** (-0.57,-0.36)
**RACE/ETHNICITY**						
Non-Hispanic Black	0.00 (-0.20,0.20)	-0.12 (-0.40,0.15)	-0.41 (-0.66,-0.16)	-0.69 (-1.04,-0.33)	-0.79 (-0.91,-0.67)	-0.76** (-0.93,-0.60)
Hispanic	0.34** (0.17,0.51)	0.00 (-0.23,0.02)	0.06 (-0.14,0.04)	-0.22 (-0.48,0.03)	-0.27 (-0.39,-0.14)	-0.53** (-0.65,-0.40)
Other	-0.25* (-0.48,-0.03)	-0.33* (-0.66,-0.01)	-0.21 (-0.45,0.04)	-0.35* (-0.66,-0.04)	-0.68** (-0.83,-0.53)	-0.77** (-0.96,-0.57)
**EDUCATION**						
H.S. Graduate	-0.15 (-0.40,0.10)	-0.26 (-0.55,0.04)	-0.31* (-0.55,-0.06)	-0.61** (-0.89,-0.33)	-0.15** (-0.26,-0.05)	-0.27** (-0.39,-0.14)
Some College or Technical Training	-0.24* (-0.49,-0.00)	-0.74** (-1.03,-0.44)	-0.33** (-0.59,-0.08)	-0.84** (-1.14,-0.55)	-0.15** (-0.26,-0.05)	-0.54** (-0.67,-0.41)
College & Post Baccalaureate	-0.24 (-0.62,0.14)	-1.00** (-1.58,-0.42)	-0.50* (-0.95,-0.08)	-1.66** (-2.37,-0.94)	-0.44* (-0.84,-0.03)	-0.78** (-1.34,-0.21)
Constant	0.21 (-0.08,0.50)	-0.58 (-0.93,-0.23)	0.72 (0.40,1.05)	-0.10 (-0.47,0.28)	0.89 (0.73,1.05)	0.26 (0.09,0.44)
**TOTAL OBS.**	8,356	8,355	4,226	4,223	15,386	15,356

Reference categories: Gender: male, Race/ethnicity: non-Hispanic White, Education status: Still in H.S. or did not complete H.S.

^a^Confidence intervals as computed for S.E.’s adjusted for sampling weights.

^b^Confidence intervals are computed from S.E.’s adjusted for sampling weights and sampling within strata.

*p<0.05

**p<0.01.

For past 30-day smoking, all three modality-specific regressions consistently estimate a similar adjusted gender effect, indicating a lower prevalence among females compared with males (p<0.01). The coefficients for minorities in the telephone and in-person CAI surveys are consistently and significantly lower than non-Hispanic Whites (p<0.05), except for Hispanics surveyed via telephone (coef. = 0.06, p = 0.09). By contrast, statistically significant racial/ethnic differences in past 30-day smoking estimates in the online surveys are restricted to significantly lower rates for non-Hispanic Others compared with non-Hispanic Whites (coef. = -0.33, p<0.05). Data from all three survey modalities yielded significant educational effects. Regardless of modality, all surveys found a strong dose-response relationship between reduced past 30-day smoking and higher educational attainment.

## Discussion

Estimates of smoking prevalence across six national probability-based surveys of young adults age 18–21 varied significantly by survey mode. The in-person CAI surveys consistently yielded the highest estimates for ever and past 30-day cigarette smoking while online surveys yielded the lowest. In contrast, the phone surveys yielded estimates in between, but significantly different than the higher in-person CAI and lower online surveys estimates. The modality differences we found held for the sample overall, males and females, and non-Hispanic Whites. However, among minority groups (Blacks, Hispanics, and Others), there were few differences in rates across modalities. The findings overall were in contrast to our hypothesis that smoking estimates from the online survey would be higher than the other modalities due to increased anonymity. Further, while relative differences in the likelihood of smoking among subgroups were consistent across survey modes for gender and education groups, the relative likelihood of smoking among minority groups compared with non-Hispanic Whites varied across modes.

Variation in smoking prevalence rates by survey modality may be due to several factors. Over- or underestimation of smoking can occur as a result of coverage bias related to sampling frames. Samples based on online ABS frames have been found to overrepresent non-Hispanic Whites and those with higher education.[16] Indeed, we found the ABS online samples in this study overrepresented Whites and women. Given generally higher rates of smoking among non-Hispanic Whites relative to non-Hispanic Blacks and Hispanics and lower smoking among women compared with men, overcoverage of these populations in the online sample would likely have a mixed effect on smoking prevalence estimates. Alternatively, higher education or income in the ABS sample compared with national estimates could be driving lower prevalence rates. Findings here are somewhat counter to studies comparing national smoking prevalence estimates from online ABS panels and other sampling frames.[5, 17] McMillen et al.[17] found similar rates of adult current smoking prevalence across four national survey frames, including a probability-based online frame, a dual RDD plus probability based online frame, and two area-based sampling household interviews.[17] Yeager et al.[5] also obtained similar estimates in adult smoking prevalence in an RDD versus probability-based online study, and reported that the probability-based online sample had the best sample composition and self-report accuracy combined.[5]

Question wording may have also contributed to significant differences in smoking estimates. While we attempted to minimize wording differences by selecting only surveys that introduced smoking with the “ever use” question (vs. 100 cigarettes smoked), minor differences in question wording for both ever and past 30-day use remained. In assessing wording differences within mode for a web survey among young adults, McCabe et al.[18] found similar rates of substance use across surveys with equivalent wording, but significant differences in rates with minimal differences in question structure, such as adding “don’t know” or “refuse” to the response categories or slightly altering a skip pattern. This effect was consistent across gender and race/ethnic groups.[18] Rodu et al.[19] found higher smoking prevalence rates for NSDUH surveys using the past 30-day smoking ítem following 100+ cigarettes as the ‘ever use’ critiera compared with the everyday/some day ítem following the 100+ cigarettes criteria in the National Health Interview Survey. Findings suggested that the past 30-day ítem may capture more some day smokers with lower rates of smoking than the everyday/some day question, particularly among young adults. Johnson et al.[20] found that “select-all-that apply” questions to assess ever and past 30-day tobacco prevalence among young adults may underestimate prevalence as estimates from such questions may be biased downward.[21] The YAC online survey in this study used the ‘select-all-that-apply’ question format, while the TLC used a separate breakout question with an image, which is a question type that produced higher estimates in the Johnson[20] study. Nonetheless, estimates for these two surveys were similar, suggesting other factors may have contributed to the lower rates relative to other modes.

Another factor that can contribute to different estimates in certain behaviors is social desirability effects. Individuals may underreport some behaviors due to social disapproval or illegality of the behavior. Reviews of social desirability effects in smoking self-report among adults have been mixed,[22–25] although the most recent studies have found that self-reported smoking tends to be somewhat underestimated when compared to assessment of smoking using biomarkers such as saliva cotinine for the presence of tobacco. [25] Research has found that social desirability bias in smoking prevalence estimates are stronger for phone response than in person, with in-person reports generally higher than phone reports.[26–28] This pattern was found for males, females and non-Hispanic Whites in this study. Yet self-administered surveys such as online surveys are generally considered to reduce social desirability bias and improve reports of sensitive behaviors compared with interviewer-administered surveys [1, 13, 14], (although these effects can be somewhat inconsistent [29]), which would predict that online estimates would be higher as we hypothesized. However, this was not the case for males, females and non-Hispanics Whites for whom online estimates were the lowest. It may be the case that other factors, such as differences in sample coverage discussed above or social desirability biases working differently than hypothesized, might be stronger factors in the smoking estimate patterns found in this study, at least for these populations.

The pattern of smoking estimates across surveys was different for minority groups than by gender and education in that estimates did not consistently differ by mode. It is possible that social desirability effects played a role for these groups, resulting in lower reports of smoking in the in-person and phone surveys for minorities, and more accurate reporting online, thus countering the general pattern of effects across the surveys. Research has found higher substance use non-disclosure among minorities and a reduction in non-disclosure when survey conditions are perceived to be more anonymous[30–34], which may be the case with the online surveys. However, updated research is needed on this topic.

Social desirability effects among minorities may have also contributed to differences in the relative likelihood of ever or past 30-day smoking across subgroups by mode. In the tobacco literature, it is well-known that females, higher educated individuals, Hispanics, and Blacks are less likely to smoke compared with males, lower educated individuals, and non-Hispanic Whites, respectively.[35] In this study, we found the expected pattern by gender and education across all modes. The expected pattern varied by race/ethnicity across modes. The likelihood of smoking was consistently lower among minorities compared with non-Hispanic Whites, as expected, for the household and phone modes. In contrast, online surveys elicited smoking rates for Blacks and Hispanics that were similar or higher relative to non-Hispanic Whites.

Finally, modality differences may arise because of population differences in participation rates. Even when households are offered computers and free internet access, participation among low SES and minority populations may be reduced if they experience less comfort interacting with computers and the internet.

This study has both strengths and limitations. While we selected surveys with similar tobacco use questions and only examined estimates within the same age group to minimize differences, other factors may be responsible for the differences in prevalence estimates. Further, the use of national samples minimized the possibility of differences attributable to characteristics of local samples. It is also possible that a portion of what we have interpreted as modality effects may be due to systematic differences related to organization administrating the survey. Each survey type was administered by the same organization (i.e., the in-person CAI surveys by the SAMSHA, the telephone surveys by CDC and the online surveys by Truth Initiative). Thus further research needs to examine results from surveys with modality types from different organizations to better disentangle potential modality effects from organization-specific factors. The greatest strength of this study is the rigor of the design, methodology, and questions items for each of the surveys included in the sample.

It is also possible that modality effects may be linked to lifecourse processes, such as transitions from school to work that are specific to the narrow age range of this study. Survey modalities may have different consequences for older adults who have aged out of the early smoking initiation process. Different work/life patterns of early young adults and older adults as well as varying levels of sensitivity regarding reporting certain behaviors such as smoking may also differ over the lifecourse and across cohorts.

While our focus here is on variation in estimates of smoking prevalence, researchers from all fields must give increased attention to new technologies that alter modes of data collection and contribute to variation in health risk behavior estimation. Research is needed to evaluate differences in health behavior estimates across surveys due to variation in population and subpopulation sample coverage, modal effects, social desirability bias and other factors. Estimates of the prevalence of a behavior are often used to establish target populations for health promotion and disease prevention interventions. As survey research technologies evolve, understanding differences in behavioral estimates across surveys is increasingly important to ensure accuracy of estimates and inform policy and interventions.
